# Cognition and action: a latent variable approach to study contributions of executive functions to motor control in older adults

**DOI:** 10.18632/aging.203239

**Published:** 2021-06-24

**Authors:** Caroline Seer, Justina Sidlauskaite, Florian Lange, Geraldine Rodríguez-Nieto, Stephan P. Swinnen

**Affiliations:** 1 Movement Control and Neuroplasticity Research Group, Department of Movement Sciences, KU Leuven, Flanders, Belgium; 2 KU Leuven Brain Institute (LBI), KU Leuven, Flanders, Belgium; 3 Behavioral Engineering Research Group, KU Leuven, Flanders, Belgium

**Keywords:** aging, motor control, bimanual coordination, executive functions, structural equation modeling

## Abstract

Aging is associated with profound alterations in motor control that may be exacerbated by age-related executive functioning decline. Executive functions span multiple facets including inhibition (suppressing unwanted response tendencies), shifting (switching between cognitive operations), and updating (managing working memory content). However, comprehensive studies regarding the contributions of single facets of executive functioning to movement control in older adults are still lacking. A battery of nine neuropsychological tasks was administered to *n* = 92 older adults in order to derive latent factors for inhibition, shifting, and updating by structural equation modeling. A bimanual task was used to assess complex motor control. A sample of *n* = 26 young adults served as a control group to verify age-related performance differences. In older adults, structural equation models revealed that performance on the most challenging condition of the complex motor task was best predicted by the updating factor and by general executive functioning performance. These data suggest a central role for working memory updating in complex motor performance and contribute to our understanding of how individual differences in executive functioning relate to movement control in older adults.

## INTRODUCTION

Aging is associated with a motor functioning decline that must be addressed to promote healthy and active living throughout the lifespan [1, 2]. Evidence from dual-task paradigms and findings of cortical hyper-activation from functional neuroimaging suggest motor control to become less automated and more effortful during aging [1–7]. Specifically, older adults have been suggested to engage higher-order cognition (executive functions) to a larger extent than younger adults when performing complex motor tasks, possibly reflecting the recruitment of generic brain regions to support motor performance [3–5, 8–10]. Hence, intact executive functioning may be particularly crucial for older adults when performing complex motor control tasks, such as bimanual coordination [6, 11, 12]. Indeed, age-associated bimanual coordination changes have been found in various paradigms [1]. Similar to motor performance, executive functions decline during aging [13–16]. Such executive functioning decline might exacerbate age-related difficulties in motor control and should therefore be taken into account when investigating age-related changes in motor functions.

Executive functions are a set of dissimilar capacities rather than one unitary ability. Three key facets of executive functioning are inhibition (i.e., suppressing unwanted response tendencies), shifting (i.e., switching between cognitive operations), and updating (i.e., managing working memory content) [8, 17, 18]. Despite this heterogeneity of executive functions and their hypothesized link with motor abilities especially during aging, few studies have addressed how individual differences in multiple facets of executive functioning are associated with complex motor control in older adults. However, such a multifaceted approach to executive functioning (i.e., one that takes into account multiple dissimilar domains of executive functioning) is crucial to differentiate between the contributions of distinct executive functions to individual differences in motor performance. Bangert and colleagues reported bimanual circle-drawing performance to be related to older adults’ working memory (assessed as backward digit span) [19]. This relationship was restricted to the most challenging task condition, and no significant relationship was found with inhibition and shifting performance. Similarly, Corti and colleagues examined associations between executive functioning and fine motor control (assessed by the Purdue Pegboard Test) in older adults across several executive functioning domains (working memory, set-shifting, planning) [20]. They found performance on a single task assessing planning abilities (a higher-order executive function [8]) to be the most consistent predictor of motor performance in older adults across unimanual and bimanual conditions.

Taken together, the available evidence regarding the link between distinct facets of executive and motor control in older adults is still scarce and fragmented, and especially studies taking into account the multifaceted nature of executive functioning are lacking. In addition, the available studies address contributions of executive functions to complex motor control on the level of single tasks (i.e., reporting the performance on one particular task as an indicator for the corresponding executive function). This represents a critical methodological limitation because every cognitive laboratory task is ‘impure’, i.e. necessarily captures variability that is unspecific to the function under investigation (e.g., visual processing), hampering both reliability and generalizability. This task-impurity problem can be mitigated by the use of multiple tasks for every cognitive domain under investigation. The shared variance among tasks representing the same function can then be modeled as latent factors [9, 17, 18]. Such latent variable approaches are therefore particularly suitable for the assessment of executive functions, but have not been applied to study the individual contributions of dissimilar executive functioning domains to complex motor control in older adults [13, 16–18, 21–25].

**Figure 1:**
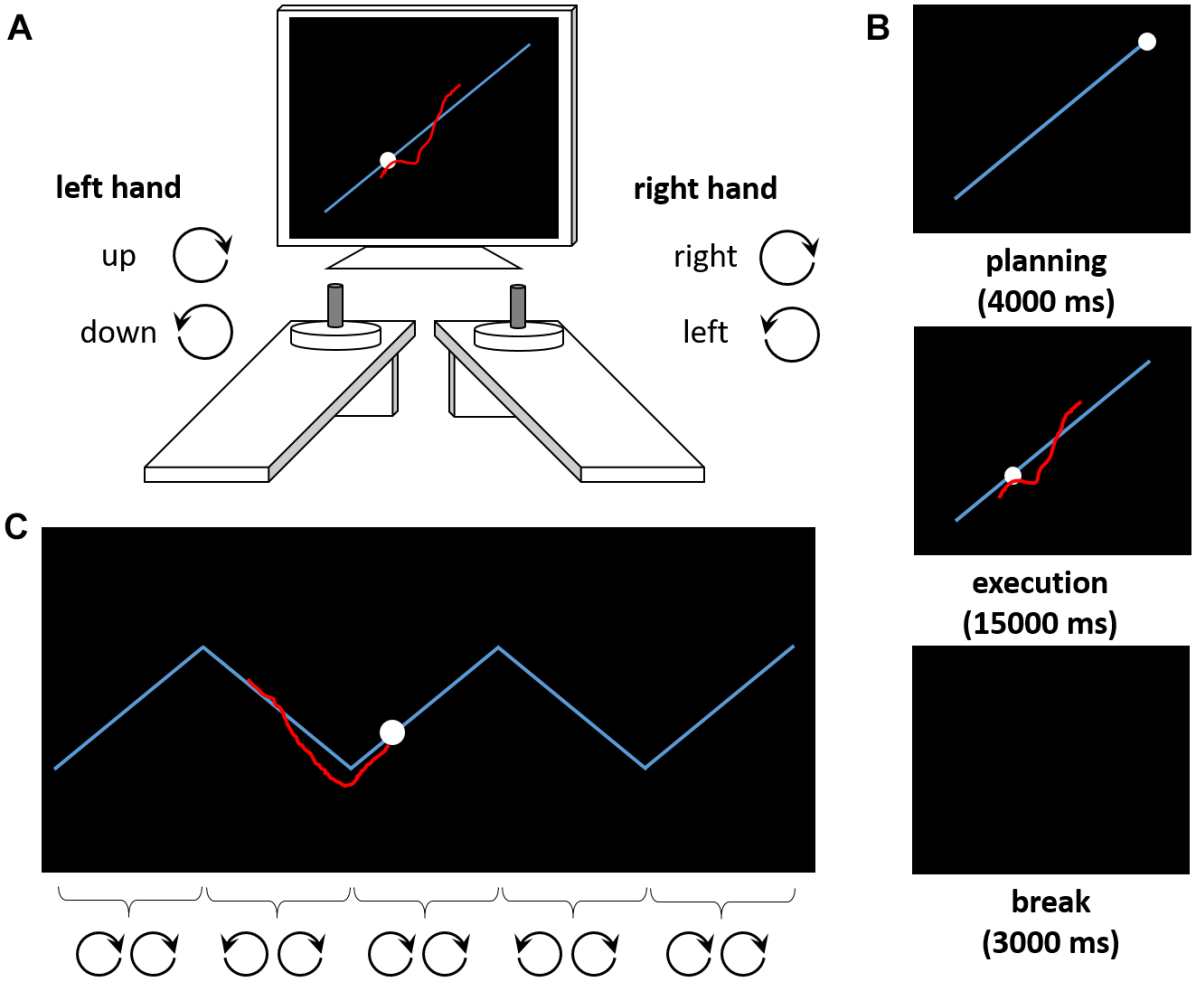
**The Bimanual Tracking Task (BTT).** (**A**) The task setup consists of two dials placed in front of a computer screen. Participants are asked to rotate both dials simultaneously to track a moving dot along a target line. Rotating the left dial clockwise (counterclockwise) causes the red cursor to move upward (downward) along the Y-axis, whereas rotating the right dial clockwise (counterclockwise) causes the cursor to move to the right (left) along the X-axis. (**B**) Exemplary trial sequence. After a planning phase of 4000 ms, the movement is executed (15000 ms). A break of 3000 ms precedes the next trial. (**C**) Exemplary trial from the zigzag condition. The target trajectory requires periodic switches in the rotation of one (here: left) hand, whereas the other (here: right) hand should continue its movement. For illustration purposes, the correct rotation directions for both hands are indicated for each segment of the zigzag trajectory here.

Here, we investigate how distinct facets of executive functioning contribute to complex movement control in older adults. For this purpose, we use a bimanual tracking task (BTT [26]) which is sensitive to age-associated changes [27–32]. The BTT requires participants to perform rotational movements with both hands simultaneously (Figure 1A). The complex BTT-condition requires one hand to perform periodic switches of the rotational direction whilst the other hand needs to maintain a continuous rotational movement (Figure 1C). There are strong conceptual reasons to expect contributions of executive functions to BTT-performance as the BTT and executive functioning paradigms share critical task demands. Specifically, conceptual overlap with response-inhibition tasks involves selectively suppressing unwanted movements of one hand while continuing the other hand’s movement. Similarly, the BTT converges with shifting tasks in that performers need to shift attention from keeping both hands moving to reversing the movement of one hand selectively. Finally, overlaps with working memory tasks involve repeated updating of the prevailing movement pattern and monitoring the two hand movements simultaneously whilst comparing performance to the task goal (here: comparing the position of a cursor to a target on the screen).

These conceptual overlaps justify to assume that inhibition, shifting, and updating are all substantially related to motor performance. However, to determine if any of these executive functions contributes particularly strongly to motor control, they need to be studied simultaneously (i.e., in a multifaceted approach) and with the necessary attention toward the problems arising from task impurity (i.e., in a latent variable approach). Such comprehensive data—taking into account both the multifaceted nature of executive functioning and the task-impurity problem—are currently lacking. This study is the first to examine the contributions of multiple facets of executive functioning to complex motor performance in older adults by using latent variable modeling, thereby creating a basis for a more detailed understanding of the link between executive abilities and movement control in aging.

## MATERIALS AND METHODS

### Participants

One hundred and thirteen older adults (≥ 60 years) were recruited from the area of Leuven. We chose to retain only complete datasets for analysis and therefore excluded the following cases: two participants who opted out of the complex motor task, sixteen participants for insufficient adherence to task instructions on at least one neuropsychological task (as evidenced by performance that was indistinguishable from chance level; see Supplementary Materials), one participant because of recording failure, two participants because testing was aborted after one session (see Supplementary Materials) due to COVID-19 containment procedures. The effective sample size was *n* = 92 older adults (55 female, 37 male; 4 left-handed, 11 ambidextrous, 77 right-handed [33, 34]). Normal (or corrected-to-normal) vision was required for inclusion. Exclusion criteria were current intake of psychoactive medication and the presence of psychiatric/neurological disorders, upper limb injury that would have interfered with BTT-completion, and/or contraindications for magnetic resonance imaging (this study was part of a larger project; neuroimaging results will be reported elsewhere). None of the participants showed signs of mild cognitive impairment on the Montreal Cognitive Assessment (MoCA; range: 24-30 [35, 36]).

Thirty-three young adults were recruited to verify the presence of age-associated executive and motor performance differences. Seven participants were excluded for insufficient adherence to task instructions on at least one neuropsychological task (see Supplementary Materials). The effective sample size for young adults was *n* = 26 (16 female, 10 male; 4 left-handed, 4 ambidextrous, 18 right-handed). Apart from the targeted age range (18-40 years), inclusion criteria and procedures were identical for the groups. Table 1 displays the sample characteristics.

**Table 1:** Sociodemographic characteristics and background information of the study sample.

	**Young adults (*n* = 26)**	**Older adults (*n* = 92)**	***d* [95%-CI]**
	***M* (*SD*)**	***M* (*SD*)**	
Age (years)^a^	23.35 (4.47)	67.99 (4.61)	9.74 [8.41; 11.08]
Education (years)	18.98 (1.91)	18.13 (2.67)	-0.34 [-0.79; 0.11]
MoCA	28.96 (1.48)	27.73 (1.77)	-0.72 [-1.17; 0.27]
PPVT-III-NL	111.19 (8.00)	109.35 (8.73)	-0.22 [-0.66; 0.23]
BSI-18 Global Severity Index	5.73 (4.34)	3.79 (4.90)	-0.40 [-0.85; 0.04]
Depression	1.62 (1.63)	0.82 (1.65)	-0.49 [-0.93; -0.04]
Anxiety	2.50 (2.21)	1.71 (2.63)	-0.31 [-0.75; 0.13]
Somatization	1.62 (2.40)	1.27 (1.85)	-0.17 [-0.61; 0.27]
MBQ^b^ Total	8.32 (1.24)	8.30 (1.31)	-
Work / Household	2.16 (0.53)	1.93 (0.35)	-
Sport	3.13 (0.73)	3.15 (0.67)	-
Leisure	3.03 (0.59)	3.21 (0.64)	-
IPAQ Total	4218.94 (3703.67)	5210.64 (4513.12)	0.23 [-0.21; 0.67]
Work	935.10 (2009.01)	772.85 (2643.13)	-0.06 [-0.50; 0.38]
Transport	1401.98 (1146.70)	1193.84 (1259.55)	-0.17 [-0.61; 0.27]
House	481.92 (1009.80)	1886.90 (2397.35)	0.65 [0.20; 1.09]
Leisure	1399.94 (1556.30)	1357.04 (1683.82)	-0.03 [-0.47; 0.41]
Sitting	2905.20 (1183.25)	2232.81 (1046.47)	-0.62 [-1.08; -0.16]
RAND-36			
Physical Functioning	98.46 (3.68)	87.39 (13.27)	-0.93 [-1.39; -0.48]
Social Functioning	91.35 (13.59)	93.61 (13.17)	0.17 [-0.27; 0.61]
Role Limitations (Physical)	94.23 (14.68)	89.95 (26.22)	-0.18 [-0.62; 0.26]
Role Limitations (Emotional)	88.46 (26.57)	96.74 (12.16)	0.51 [0.06; 0.95]
Mental Health	74.15 (15.60)	81.13 (12.53)	0.53 [0.08; 0.97]
Vitality	64.42 (17.96)	74.57 (12.87)	0.72 [0.27; 1.17]
Pain	87.76 (12.66)	83.63 (17.73)	-0.25 [-0.69; 0.20]
General Health Perception	73.08 (13.72)	70.22 (14.52)	-0.20 [-0.64; 0.24]
Health Change	55.77 (19.12)	51.36 (16.31)	-0.26 [-0.70; 0.18]

*Note*: MoCA = Montreal Cognitive Assessment, higher scores indicate better cognitive status [36]; PPVT-III-NL = Dutch version of the Peabody Picture Vocabulary Test, higher scores indicate higher crystallized intelligence [46, 47]; BSI-18 = Brief Symptom Inventory, 18-item version, higher scores indicate higher symptom severity [48]; MBQ = Modified Baecke Questionnaire, higher scores indicate higher levels of physical activity [49]; IPAQ = International Physical Activity Questionnaire, higher scores indicate higher levels of physical activity (except for “sitting”, where higher scores indicate lower levels of physical activity) [50]; RAND-36 = Short Form Health Survey, higher scores indicate better health-related well-being [51, 52]. ^a^ Effective age range: 18-37 years (young adults), 60-85 years (older adults). ^b^ Age-specific versions were used for older and young adults [53]. The scale “work” (“household”) applies to young adults (older adults) only.

The study was reviewed and approved by the Ethics Committee Research UZ/KU Leuven. All participants gave written informed consent to participate and were offered € 100 as compensation. The dataset and code are available on https://www.osf.io/5v2rz.

### Procedure

### Neuropsychological tasks

Executive function assessment followed the protocol of Friedman and colleagues, with minor modifications [37]. Inhibition, shifting, and updating were each examined by three well-established and validated tasks (inhibition: antisaccade task (AT) [37–40], number-Stroop task (NST) [37, 41–43], stop-signal task (SST) [37, 39, 44, 45]; shifting: category-switch task (CAST) [37, 39, 54, 55], color-shape task (COST) [37, 39, 42, 56], number-letter task (NLT) [37, 39, 57]; updating: digit-span task (DST) [42, 58], keep track task (KTT) [37, 39, 59], spatial 2-back task (STT) [37, 39, 60]; see Supplementary Materials for details on timing and trial numbers). Neuropsychological tasks were programmed and controlled by OpenSesame version 3.2.6 [61]. Responses were collected on a standard QWERTY computer keyboard.

### Motor task

Complex motor control was assessed using the BTT [26], which was controlled by LabView 2016 (National Instruments, Austin, TX). Participants tracked a moving dot on a target line on the computer screen by bimanually rotating two dials at a prescribed frequency. Clockwise (counterclockwise) rotations with the right hand caused the cursor to move to the right (left) on the computer screen. Clockwise (counterclockwise) rotations with the left hand caused the cursor to move upward (downward; Figure 1A). In the ‘straight’ condition, the target trajectory was a diagonal line (i.e., both dials should be rotated at the same speed in a constant direction, Figure 1B). In the ‘complex’ condition, the target trajectory was a zigzag line, with abrupt changes of direction [28, 32] (i.e., rotation direction in one hand should be maintained, whereas rotation direction of the other hand should be adjusted whenever the target dot changed its direction on the trajectory, Figure 1C; see Supplementary Materials for details on timing and trial numbers). The outcome measure was the average accuracy of the tracking performance across trials (see Supplementary Materials for calculation and technical details). Briefly, accuracy scores reflect how well the cursor is moved on top of or parallel to the target line at the same speed as the target dot. Accuracy decreases as a result of (a) too fast or too slow cursor movements, (b) movements away from the target line or in the wrong direction, or (c) cutting corners in the ‘zigzag’ condition.

### Data analysis

The neuropsychological data were processed in SPSS 26 (IBM, Armonk, NY) to derive the outcome measures (see Supplementary Materials) [37]. BTT data were analyzed in Matlab 2019b (MathWorks, Natick, MA). Data analysis was performed in R 4.0.2 [62] in RStudio 1.3 (RStudio, Boston, MA), relying on the lavaan package version 0.6-7 [63] for structural equation modelling.

### Age-associated differences in executive functions and motor performance

One-sided independent samples *t*-tests were used to verify the presence of age-associated differences between young and older adults in neuropsychological and motor tasks.

### Relations between executive functions and motor performance in older adults

Structural equation models were defined to probe relations between complex motor control and inhibition, shifting, and updating. The outcomes of AT, NST, and SST were used as indicators for a latent inhibition factor. Similarly, the outcomes of CAST, COST, and NLT were used as indicators of shifting, and the outcomes of DST, KTT, and STT were used as indicators of updating. These latent factors were modeled as predictors of accuracy on the complex BTT-condition in older adults.

We first modeled inhibition, shifting, and updating as dissociable, but correlated factors (“correlated factors model”) [17]. Next, we ran a model where a common executive functioning factor (“Common EF”) accounts for shared variance across all indicators (“bifactor model”). For this model, shifting-specific and updating-specific factors were created based on the respective indicator tasks. These specific factors were modeled orthogonally to each other and to the Common EF factor, hence representing unique variance [18, 37]. Note that an inhibition-specific factor is not typically found when testing young adults, with mixed findings in older adults [9, 13, 16, 18, 22–25]. Hence, we also ran a model including an inhibition-specific factor to explore its presence in this sample. Finally, to assess whether the results were specific to the complex BTT-condition, we re-ran the models using the simple condition as a dependent variable.

## RESULTS

### Age-associated differences in executive functions and motor performance

We found executive and motor performance differences between young and older adults on all except two tasks (Table 2). On the COST, switch costs were numerically higher in older compared to young adults, but not significantly different. On the NST, older adults showed less susceptibility to response conflict compared to young adults. This can be explained by generally slowed reaction times (RTs) in older adults, which may have masked condition differences (congruent: *M* = 1003.35 ms; incongruent: *M* = 1092.14 ms), whereas RTs showed stronger modulations as a function of condition in young adults (congruent: *M* = 630.69 ms; incongruent: *M* = 800.81 ms). Hence, the NST may not be a suitable indicator of inhibition in this study, at least not for older adults, and should be interpreted cautiously.

**Table 2:** Outcome measures for the executive and motor tasks for young (n = 26) and older adults (n = 92).

**task**	***M***	***SD***	**min**	**max**	**skewness**	**kurtosis**	**group difference**
AT (proportion of correct responses in antisaccade blocks)							*t*(116) = 7.15*p* < .001*d* = 1.59[1.10; 2.07]
young	0.76	0.14	0.40	0.95	-0.74	-0.01	
older	0.53	0.14	0.21	0.89	0.31	-0.09	
NST (median RTs for correct responses for incongruent minus congruent trials)							*t*(116) = 3.44*p* > .999*d* = 0.76[0.31; 1.22]
young	170.12 ms	72.67	-14.00	292.00	-0.52	-0.10	
older	88.78 ms	113.86	-198.00	340.00	-0.26	-0.41	
SST (stop-signal RT)							*t*(116) = -5.66*p* < .001*d* = -1.26[-1.73; -0.79]
young	222.96 ms	26.35	173.00	279.50	-0.11	-0.87	
older	272.50 ms	42.28	140.08	404.44	-0.45	2.03	
CAST (difference of median RTs between switch and repeat trials)							*t*(116) = -3.74*p* < .001*d* = -0.83[-1.28; -0.38]
young	173.92 ms	99.89	-11.50	350.00	0.14	-1.00	
older	348.30 ms	231.39	-62.50	1068.80	0.97	0.75	
COST (difference of median RTs between switch and repeat trials)							*t*(116) = -1.08*p* = .141*d* = -0.24[-0.68; 0.20]
young	290.89 ms	135.81	65.00	673.00	0.75	0.39	
older	369.79 ms	363.94	-489.00	1631.33	1.22	2.09	
NLT (difference of median RTs between switch and repeat trials)							*t*(116) = -2.18*p* = .016*d* = -0.48[-0.93; -0.04]
young	306.26 ms	239.67	18.00	1042.14	1.26	1.32	
older	416.68 ms	225.21	-53.50	1119.31	0.81	0.70	
DST (total number of correct trials)							*t*(116) = 5.88*p* < .001*d* = 1.31[0.83; 1.78]
young	15.12	4.67	5.00	28.00	0.38	0.66	
older	10.37	3.29	5.00	20.00	0.79	0.38	
KTT (proportion of correctly recalled words)							*t*(116) = 5.73*p* < .001*d* = 1.27[0.80; 1.74]
young	0.78	0.09	0.63	0.98	0.39	-0.16	
older	0.65	0.11	0.32	0.95	-0.22	0.34	
STT (proportion of correct responses)							*t*(116) = 5.35*p* < .001*d* = 1.19[0.72; 1.65]
young	1.33	0.08	1.13	1.49	-0.40	0.49	
older	1.20	0.12	0.91	1.40	-0.33	-0.53	
BTT-simple (percent coverage of the target line)							*t*(116) = 4.58*p* < .001*d* = 1.02[0.56; 1.48]
young	89.19 %	2.40	82.13	92.10	-1.04	0.60	
older	84.98 %	4.50	73.14	91.92	-0.76	-0.02	
BTT-complex (percent coverage of the target line)							*t*(116) = 8.09*p* < .001*d* = 1.80[1.30; 2.29]
young	76.09 %	4.55	65.76	84.52	-0.28	-0.29	
older	62.88 %	7.95	45.83	82.51	-0.12	-0.33	

*Note:* Values for the neuropsychological tasks are displayed after between-subjects trimming and transformation (see Supplementary Materials). Group differences are tested one-sided, hypothesizing better performance in young as compared to older adults. Confidence intervals indicate 95% confidence intervals for *d*.

### Relations between executive functions and motor performance in older adults

**Figure 2:**
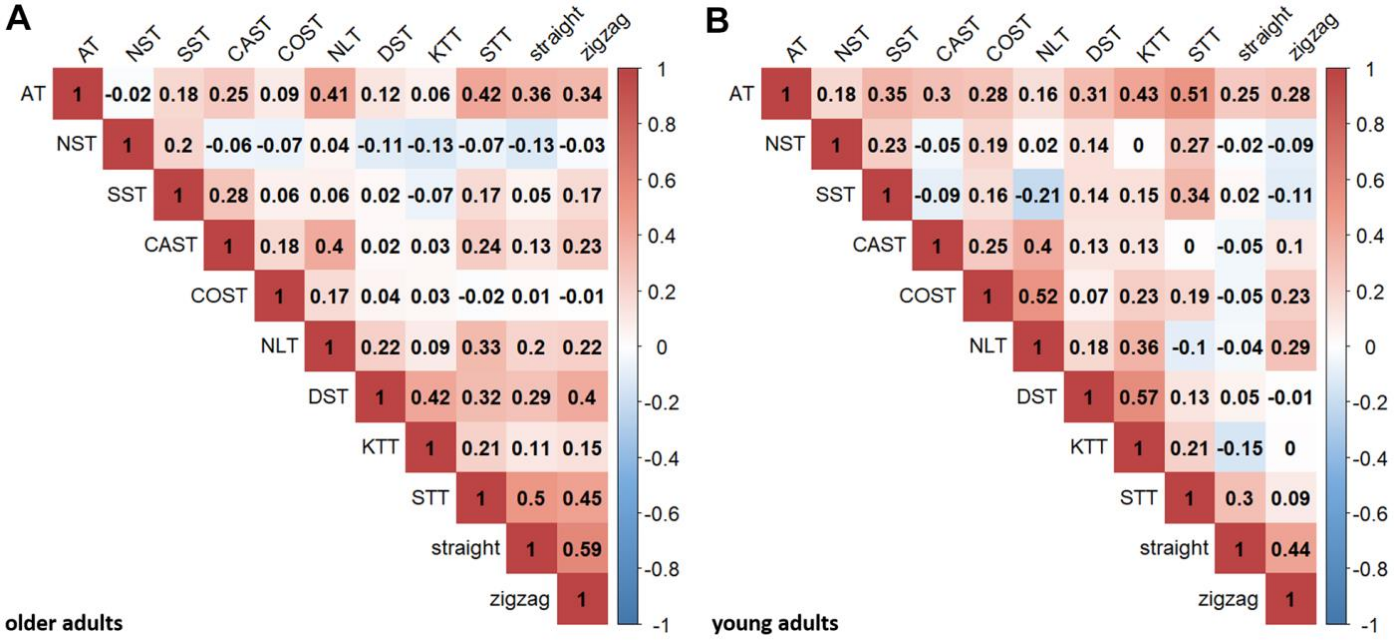
**Pearson correlations between executive and motor tasks.** Pearson correlation coefficients are shown for older (**A**) and young (**B**) adults for descriptive purposes (critical *r*-value for *p* < .05, uncorrected: .205 (older adults), .389 (young adults); critical *r*-value for *p* < .000909, Bonferroni-corrected (.05/55): .341 (older adults), .612 (young adults)). AT, NST, SST represent inhibition; CAST, COST, NLT represent shifting; DST, KTT, STT represent updating. “straight” and “zigzag” indicate the respective BTT-conditions. All tasks were transformed so that higher scores indicate better performance.

Figure 2A displays Pearson correlations between the neuropsychological and motor tasks for the present sample of older adults. The neuropsychological tasks were mostly positively intercorrelated, with the exception of NST. The BTT correlated with various neuropsychological tasks. For comparison, Figure 2B displays zero-order correlations for the present sample of young adults.

**Figure 3:**
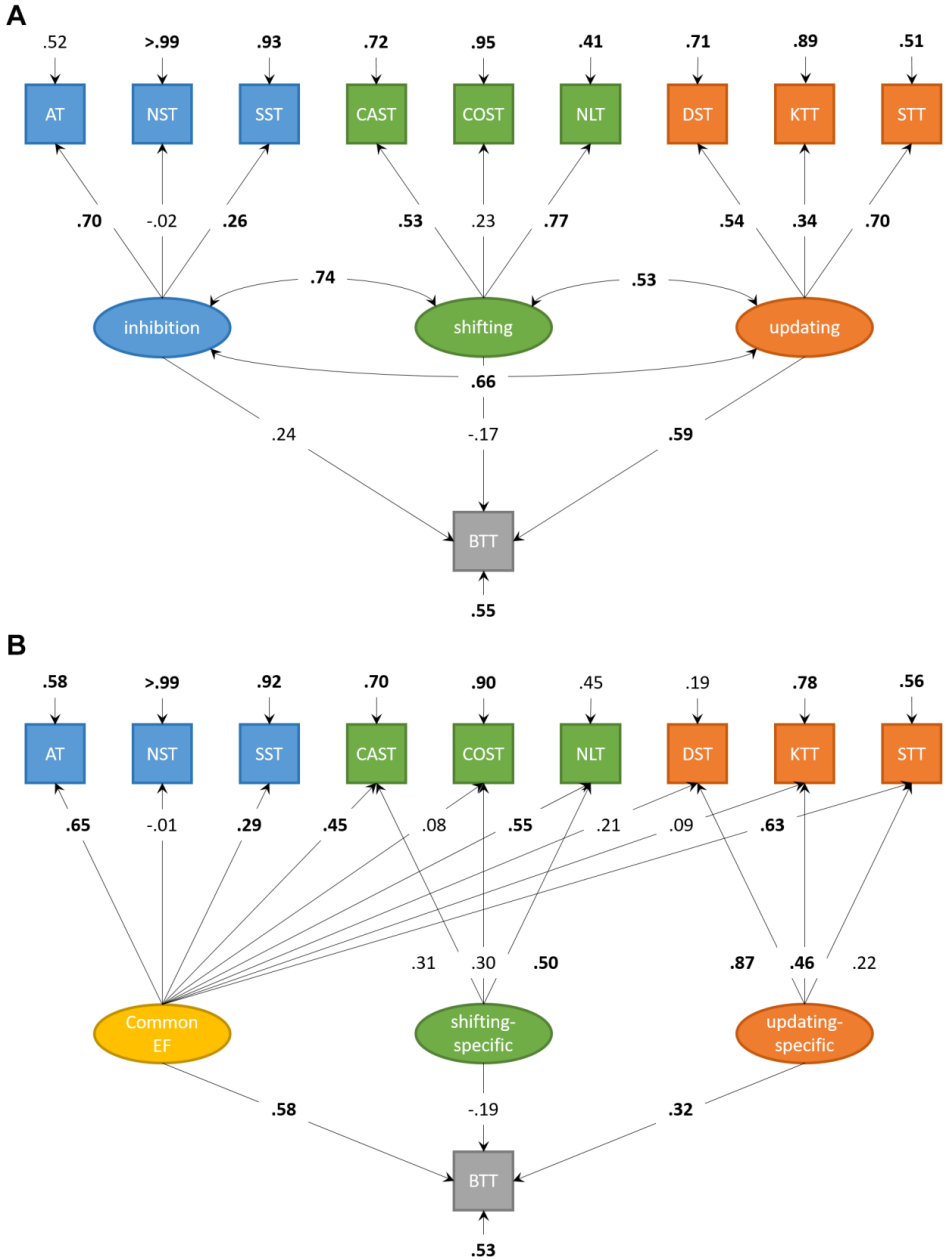
**Structural equation models for executive functions and motor performance in older adults.** (**A**) Structural equation model for correlated factors of inhibition, shifting, and updating. Updating significantly predicts performance on the complex condition of the bimanual coordination task in older adults. (**B**) Structural equation model with orthogonal factors, accounting for variance shared by all neuropsychological tasks (Common EF). Both Common EF and the updating-specific factor predict unique performance on the complex condition of the bimanual coordination task in older adults. Significant parameters are highlighted in boldface.

The correlated factors model (Figure 3A) showed acceptable fit, χ^2^(30) = 41.17, *p* = .084; CFI = .90; RMSEA = .06; SRMR = .07. The executive factors were intercorrelated (.53-.74, all *p* < .01). Updating significantly predicted complex BTT-performance, β = .59, *p* = .023, 95%-CI [0.08; 1.09]. Inhibition and Shifting did not significantly predict complex BTT-performance (β = .24, *p* = .593, 95%-CI [-0.63; 1.10], and β = -.17, *p* = .596, 95%-CI [-0.79; 0.46], respectively).

The bifactor model (Figure 3B) showed a good fit, χ^2^(27) = 24.42, *p* = .607; CFI > .99; RMSEA < .01; SRMR = .06. Common EF predicted complex BTT-performance, β = .58, *p* < .001, 95%-CI [0.33; 0.82]. The updating-specific factor, but not the shifting-specific factor (β = -.19, *p* = .289, 95%-CI [-0.54; 0.16]), predicted additional unique variance in complex BTT-performance, β = .32, *p* = .018, 95%-CI [0.06; 0.59]. Adding an inhibition-specific factor to this bifactor model (not displayed) resulted in comparable model fit, χ^2^(23) = 19.67, *p* = .662; CFI > .99; RMSEA < .01; SRMR = .05. However, this inhibition-specific factor did not explain additional unique variance in complex BTT-performance above and beyond Common EF (β = .57, *p* < .001, 95%-CI [0.32; 0.82]) and the updating-specific factor (β = .32, *p* = .021, 95%-CI [0.05; 0.59]), β = .06, *p* = .724, 95%-CI [-0.27; 0.38].

When we re-ran the models using the simple BTT-condition as a dependent variable to assess whether the results were specific to the complex BTT-condition, the correlated factors model showed comparable fit to the initial version, χ^2^(30) = 38.45, *p* = .139; CFI = .92; RMSEA = .06; SRMR = .07. Updating remained a significant predictor of motor performance, β = .57, *p* = .017, 95%-CI [0.10; 1.04]. For the bifactor model, fit indices were also comparable to the initial version, χ^2^(27) = 25.83, *p* = .528; CFI > .99; RMSEA < .01; SRMR = .06. Common EF significantly predicted performance on the simple BTT-condition, β = .61, *p* < .001, 95%-CI [0.36; 0.84]. However, in contrast to the complex BTT-condition, the updating-specific factor did not significantly predict additional variance in the simple BTT-condition, β = .20, *p* = .153, 95%-CI [-0.07; 0.47].

## DISCUSSION

This study is the first to examine the contributions of individual differences in distinct facets of executive functioning to complex motor performance in older adults by latent variable modeling. A latent updating factor predicted complex motor performance, even when accounting for shared variance with other executive tasks. This updating factor reflected variance that is specific to working memory. General executive abilities also predicted motor performance. These results reveal a unique contribution of individual differences in the ability to monitor and manipulate working memory content to motor performance in older adults, but also highlight the dependence of motor control on general executive abilities.

The relationship between updating and motor performance in older adults may be explained by the fact that the complex BTT-condition overlaps with the demands posed by the tasks subsumed under updating in that it involves a high working memory load. Specifically, to successfully follow the zigzag trajectory on the screen, participants need to monitor and control the dissimilar movements of both hands simultaneously. While one hand needs to perform regular movement switches to reverse the rotation direction, the other hand should continue its movement with minimal interruption. In addition, the cursor location needs to be compared to the target position on the screen, calling for real-time adjustments, which arguably poses significant demands on working memory. In support of this interpretation, the link between updating and motor performance did not remain significant for the simple BTT-condition without movement switches after accounting for Common EF. Our interpretation is also backed up by a previous report of correlations between challenging bimanual coordination tasks and working memory [19].

Latent factors reflecting shifting and inhibition did not significantly predict motor performance in this dataset. Shifting factors showed a numerically negative relationship with complex motor control, which is consistent with a proposed trade-off between stability and flexibility, where individuals with lower cognitive flexibility may be able to shield task performance more efficiently from external distractions compared to individuals with higher cognitive flexibility [9, 64]. Future studies could further examine this relationship in larger samples. We did not find evidence for inhibition-specific contributions to complex motor performance in this sample. In the correlated factors model, the inhibition factor showed a positive, but non-significant relationship with motor performance, which is broadly consistent with earlier reports based on findings on a single inhibition task [65]. In the bifactor model, the “inhibition”-specific factor mainly captured variability in NST (standardized factor loading: .59) and less from other inhibition tasks (AT: .02, SST: .38), and hence might not have reflected inhibition optimally. Given that earlier work did not find a separable inhibition factor at all in older adults [13, 22–24] (but see [16, 25]), future studies may further determine under which circumstances inhibition factors are distinguishable in older adults and how they contribute to motor control.

This study does not allow for a mechanistic interpretation of the link between updating and motor control in older adults [66]. However, longitudinal training studies could determine whether training-induced improvement of updating aids motor performance. If complex motor control could benefit from better updating ability (rather than merely coinciding with it), this would have important implications. First, it would allow for a more detailed understanding of the role of cognitive functions in motor control in general. Second, it would open up possibilities for designing cognitive training tools to ameliorate motor coordination in older adults.

When interpreting these data, some limitations should be considered. While the current sample size exceeds that of previous studies regarding similar research questions, it is still relatively small for structural equation modeling. This is partly due to our rigorous efforts to guarantee high data quality by implementing strict inclusion criteria based on sample characteristics and task performance. Specifically, insufficient performance on the SST led to the exclusion of a relatively high number of participants. The nature of the SST makes it challenging to avoid “waiting” for a potential stop-signal before reacting, imposing tolerance toward errors on the participant. Future studies should prevent performance-based exclusions by using an SST-version where waiting strategies are discouraged by design [67]. Moreover, the validity of the NST as an inhibition measure remains unclear in this dataset. Inspection of the data suggests that in older adults, RT differences between conditions were masked by generally slowed responding, reducing differences between congruent and incongruent conditions. This might be prevented in future work by manipulating congruency in a trial-wise, rather than block-wise manner and/or by introducing response-time pressure. While the NST did not capture much variance related to the other two inhibition tasks, the AT and SST could still be utilized as indicators of inhibition. Finally, note that the young control sample was recruited in order to verify the previously reported age-related between-groups task performance differences. Latent variable modeling of executive functioning in this group was beyond the scope of this study and would not have been possible due to sample size requirements. Age-related differences in the latent factor structure of executive functioning and their implications should be investigated in future work.

Taken together, this study sheds light on the interrelations between individual differences in multiple distinct facets of executive functioning and complex motor performance at older age. Importantly, we examined executive functioning using a latent variable approach, mitigating the limitations associated with single task assessments [17, 21]. In addition to a relation between motor performance and common executive abilities, our data suggest a specific link between older adults’ capability to monitor and update working memory content and performing complex motor actions with both hands simultaneously. These findings extend our understanding of motor decline in aging and suggest new routes for designing cognitive training tools to preserve motor control across the lifespan.

## Supplementary Material

Supplementary Methods

Supplementary Figures

Supplementary Table 1
