# Supplementary material for: Cognition and action: a latent variable approach to study contributions of executive functions to motor control in older adults
Source: Aging (Albany NY). 2021 Jun 24;13(12):15942–63. doi: 10.18632/aging.203239 (PMC8266336; doi:10.18632/aging.203239)
Supplement: Supplementary Methods [file aging-13-203239-s001.pdf]

SUPPLEMENTARY MATERIALS

Procedure

Neuropsychological tasks

Executive function assessment followed the protocol described by Friedman and colleagues, with some modifications [1]. Specifically, inhibition, shifting, and updating were each examined by three well-established and validated tasks. Neuropsychological tasks were programmed and controlled by OpenSesame version 3.2.6 [2]. Responses were collected on a standard QWERTY computer keyboard.

Inhibition

Versions of an antisaccade task (AT), a number version of the Stroop task (NST), and a stop-signal task (SST) served as measures of inhibition. In the AT [1, 3–5], participants had to avoid automatic saccades toward a salient cue appearing on the computer screen. On every trial, a central fixation cross was presented, followed by a cue (black square) on the right or on the left side of the screen (50 % probability for each side). The time interval between the fixation cross and the cue was variable, with one of nine durations between 1500 and 3500 ms at intervals of 250 ms. After a fixed duration (183 ms in the prosaccade and the third antisaccade block, 200 ms in the second antisaccade block, and 233 ms in the first antisaccade block, see below), the cue disappeared and a target stimulus was displayed, showing a number between 1 and 9. The target either appeared on the same side as the cue ('prosaccade') or on the opposite side ('antisaccade'). The target was masked after 150 ms by a black cross-hatching so that the participants would only be able to interpret the target if they executed the appropriate saccade (i.e., toward the cued side in prosaccade blocks and away from the cued side in antisaccade blocks). Participants indicated the number shown by the target by pressing the corresponding key on the number block of the keyboard, prioritizing accuracy over speed. To establish a prepotent response, the task began with a prosaccade block (18 trials, preceded by 12 practice trials). Then, participants completed three antisaccade blocks (36 trials each, preceded by 12 practice trials). Every block also contained two 'warm-up trials' that were discarded from the analyses. The outcome measure was the proportion of correct responses in antisaccade blocks.

The NST [1, 6–8] was modeled after the Stroop color-word interference task. In the number version, participants had to suppress the tendency to read out numbers from a string, but indicate its length instead. On every trial, participants saw a fixation cross (250 ms after a blank period of 750 ms), followed by a string of variable length (1-6 elements). The string remained on the screen until a response was made. Participants indicated the length of the string by pressing the corresponding key on the number block of the keyboard as fast and as accurately as possible. The task began with a 'neutral' block, where the strings consisted of asterisks (e.g., '* * *', correct response is '3') and did not induce response conflict (42 trials, preceded by 10 practice trials). Next, a block of 42 number strings was presented (preceded by 10 practices trials), where the length of the string corresponded to the displayed number ('congruent'; e.g., '3 3 3', correct response is '3'). Finally, two blocks of 42 'incongruent' trials were presented, where the length of the string never corresponded to the displayed number (e.g., '4 4 4', correct response is '3'). The outcome measure was the difference in median RTs (for correct responses) between incongruent and congruent trials.

In the SST [1, 4, 9, 10], participants needed to withhold the tendency to perform a simple categorization task, depending on the presence of a stop signal. On every trial, participants indicated whether a centrally presented green arrow pointed to the left ('z'-key) or to the right ('/'-key) as quickly and as accurately as possible ('go trial'). However, responses should be withheld whenever the arrow turned red ('stop trial'; 25 % of the trials). A staircase algorithm ensured that participants would be able to stop successfully on 50 % of the stop trials by adjusting the stop-signal delay (i.e., the time between the moment where a green arrow appears on the screen and the moment where this green arrow turns red). The initial stop-signal delay was set to 200 ms and increased or decreased by 50 ms, depending on whether the participant successfully withheld their response on the previous stop trial. The task began with 10 practice trials (only go trials) followed by a block of 50 go trials to establish a dominant response tendency. Next, it was explained that the following blocks would contain stop trials and that participants should try to withhold their responses when they saw the arrow turning red. It was stressed that slowing the responses should be avoided. After a block of 48 practice trials, participants completed three mixed blocks of 80 trials per block. The outcome measure was the stop-signal reaction time, defined as the difference between the median reaction time (RT) on go-trials (in mixed blocks) and the mean stop-signal delay (averaged across stop trials).

Shifting

Shifting was assessed using the category-switch task (CAST), the color-shape task (COST), and the number-letter task (NLT). In the CAST [1, 4, 11, 12], participants were required to switch between two tasks, the animacy task and the size task. On each trial, participants saw a target word on the screen (Dutch words for ‘bee’, ‘butterfly’, ‘frog’, ‘goldfish’, ‘alligator’, ‘elephant’, ‘lion’, ‘shark’, ‘cigarette’, ‘key’, ‘pen’, ‘snowflake’, ‘house’, ‘piano’, ‘ship’, ‘table’). A visual cue, starting 350 ms before the target word, indicated which of the two tasks had to be applied (heart for animacy task, crossed arrows for size task). For the animacy task, participants indicated whether the word described a living or a non-living thing, by pressing the ‘z’- or the ‘/’-key on the keyboard, respectively. For the size task, participants indicated whether the word described a thing that is smaller (‘z’-key) or larger (‘/’-key) than a football. Cue and target remained on the screen until a response was made. The next trial started 350 ms after the response. Errors were indicated by an auditory signal (200 ms). Participants first completed a block of 32 trials on the animacy task, followed by a block of 32 trials on the size task. Both single-task blocks were preceded by 12 practice trials, each, and included two ‘warm-up trials’ that were discarded from the analyses. Next, participants completed two blocks where both tasks were mixed in a pseudorandom manner (64 trials per block plus four ‘warm-up trials’, preceded by 24 practice trials). On 50 % of the trials, participants were required to repeat the task that they previously applied (‘repeat trial’), whereas they needed to switch to the other task (‘switch trial’) on the remaining trials. The outcome measure was the difference in median RTs between switch and repeat trials in mixed blocks.

In the COST [1, 4, 7, 13], participants were required to switch between two tasks, the color task and the shape task. On each trial, participants saw a target on the screen (red circle, red triangle, green circle, green triangle). A visual cue, starting 350 ms before the target word, indicated which of the two tasks had to be applied (letter ‘K’ for color task [‘color’ = ‘kleur’ in Dutch], letter ‘V’ for shape task [‘vorm’]). For the color task, participants indicated whether the target was red (‘z’-key) or green (‘/’-key). For the shape task, participants indicated whether the target was a circle (‘z’-key) or a triangle (‘/’-key). Cue and target remained on the screen until a response was made and the next trial started 350 ms after the response. Errors were indicated by an auditory signal (200 ms). Participants first completed a block of 24 trials on the color task, followed by a block of 24 trials on the shape task. Both single-task blocks were preceded by 12 practice trials and included two ‘warm-up trials’ that were discarded from the analyses. Next, participants completed two blocks where both tasks were mixed in a pseudorandom manner (56 trials per block plus four ‘warm-up trials’, preceded by 24 practice trials). Half of the trials were repeat trials and the other half were switch trials. The outcome measure was the difference in median RTs between switch and repeat trials in mixed blocks.

In the NLT [1, 4, 14], participants were required to switch between two tasks, the number task and the letter task. On each trial, participants saw a target on the screen, being composed of a number (2-9) and a letter (A, E, I, U, G, K, M, R). These number-letter combinations were presented in one quadrant of a box, with the position indicating which of the two tasks needed to be applied. If the pair appeared in one of the two top quadrants, participants had to attend to the number and indicated whether it was odd (‘z’-key) or even (‘/’-key). If the pair appeared in one of the two lower quadrants, participants had to attend to the letter and indicated whether it was a consonant (‘z’-key) or a vowel (‘/’-key). 350 ms before the target was displayed, the respective quadrant darkened, representing a visual cue for the task to be performed. Cue and target remained on the screen until a response was made and the next trial started 350 ms after the response. Errors were indicated by an auditory signal (200 ms). Participants first completed a block of 32 trials on the number task, followed by a block of 32 trials on the letter task. Both single-task blocks were preceded by 12 practice trials and included two additional ‘warm-up trials’ that were discarded from the analyses. Next, participants completed two blocks where both tasks were mixed in a pseudorandom manner (64 trials per block plus four ‘warm-up trials’, preceded by 24 practice trials). Half of the trials were repeat trials and the other half were switch trials. The outcome measure was the difference in median RTs between switch and repeat trials in mixed blocks.

Updating

A digit-span task (DST), the keep track task (KTT), and a spatial 2-back task (STT) were used as measures of updating. The DST [7, 15] required participants to recall strings of numbers in forward or backward order, with increasing lengths. In the first part (‘forward’), participants had to repeat the numbers in the same order as they appeared on the screen. In the second part (‘backward’), participants had to repeat the numbers in the reverse order, starting with the most recent element. They responded by typing their answer on the number block of the keyboard, prioritizing accuracy over speed. Every trial started with a fixation cross (1000 ms), then a variable number of digits were shown one by one for 1000 ms each. In both conditions, the initial trial consisted of three digits. Then, trial length was

increased by one digit after every two trials of the same length if the participant recalled at least one of the trials with the current length correctly. When a participant failed to repeat both strings, the block was terminated. The outcome measure was the total number of trials passed [15].

In the KTT [1, 4, 16], participants were asked to track up to five categories in a stream of words, recalling the last word presented for each of the categories at an unpredictable time. Each word belongs to one of six categories (animals [Dutch words for 'cat', 'dog', 'cow', 'horse', 'pig', 'sheep'], colors ['blue', 'green', 'grey', 'red', 'white', 'yellow'], countries ['England', 'France', 'Poland', 'Russia', 'Spain', 'Sweden'], fruit ['apple', 'banana', 'cherry', 'lemon', 'mango', 'melon'], metals ['cobalt', 'iron', 'tin', 'nickel', 'copper', 'zinc'], relatives ['mother', 'father', 'aunt', 'uncle', 'brother', 'sister']). On every trial, a number of target categories (2-5) was selected, and the category names were displayed at the bottom of the screen while 15-25 words (pseudo-randomly selected from all six categories) were shown to the participant for 2000 ms each. At the end of the trial, participants had to recall and type the most recent word for each target category. Two 2-category trials were given as practice trials, then 16 trials were administered, divided across four blocks, with each block containing one 2-, one 3-, one 4-, and one 5-category trial in random order. The outcome measure was the proportion of correctly recalled words across trials (unambiguously identifiable words with typing errors were counted as correct, where appropriate).

In the STT [1, 4, 17], participants were asked to judge whether a particular location on the screen had been highlighted two trials before the current one. Twelve white squares with black edges were presented on fixed locations distributed across a computer screen. In every block, every square was highlighted (i.e., turned black for 500 ms) twice, such that 24 of these 'flashes' occurred in a pseudorandom order. Flashes occurred one at a time, with 1500 ms between two flashes. For every flash, participants indicated whether the current square had been highlighted two trials before (by pressing the 'z'-key) or not ('/'-key). Errors (i.e., incorrect reactions or not reacting in time) were signaled by a 200 ms auditory signal. After a practice block of 20 flashes, participants completed six blocks (25 % 'yes'-responses per block). The outcome measure was the proportion of correct responses.

Motor task

Bimanual coordination was assessed using the bimanual tracking task (BTT [18]). Participants tracked a moving dot on a target line on the computer screen by bimanually rotating two dials at a prescribed frequency. Clockwise and counterclockwise rotations with the right hand caused the cursor to move to the right or left on the computer screen, respectively. Similarly, clockwise and counterclockwise rotations with the left hand caused the cursor to move upward or downward, respectively. In the 'straight' condition, the target trajectory was represented by a diagonal line (i.e., both dials should be rotated at the same speed in a constant direction). In the 'complex' condition, the target trajectory was represented by a zigzag line, with abrupt changes of direction [19, 20] (i.e., rotation direction in one hand should be maintained, whereas rotation direction of the other hand should be adjusted whenever the target dot changed its direction on the trajectory).

Participants were acquainted to the task in a first session (see General Procedure). Specifically, they were instructed on how to control the cursor by rotating the two dials. It was stressed that they should try to minimize the distance between cursor and target dot at all times. It was pointed out that both too slow and too fast movements would decrease the overall performance score, even when the trajectory was followed perfectly, because both too fast and too slow performance would result in increasing the distance between target and cursor. Then, participants practiced 16 simple 'straight' lines (four consecutive lines of each type). When they felt comfortable to proceed, the 'zigzag' lines were introduced. We explained that the zigzag trajectory would require one hand to perform changes in the rotational direction whereas the other hand was required to maintain its rotational direction. This was practiced on eight different zigzag lines (four horizontal, four vertical) with breaks in between. Each of these trials was repeated until the participant was comfortable to proceed to the next practice trial. In the end of the familiarization block, participants were asked to complete eight consecutive 'zigzag' lines (one of each type). This was followed by one last round of practicing, consisting of eight 'straight' (two consecutive of each type), four horizontal, and four vertical 'zigzag' lines (one consecutive of each type). At the end of the second testing session (see General Procedure), participants were re-acquainted to the task by completing eight 'straight' lines as well as two horizontal and two vertical 'zigzag' lines. Then, three blocks of BTT trials were administered, with short breaks in between: 1) 16 'straight' lines (four consecutive of each type), 2) 12 horizontal 'zigzag' lines (three consecutive of each type), and 3) 12 vertical 'zigzag' lines (three consecutive of each type).

The BTT was controlled by LabView 2016 (National Instruments, Austin, TX). Responses were recorded by sampling the cursor position at a rate of 100 Hz.

Every trial started with a display of the target trajectory on the computer screen ("planning phase", 4000 ms). The timing of the execution phase was invariant because of the fixed velocity of the target dot (15000 ms). Between two consecutive trials, there was a short break of 3000 ms.

Performance accuracy on the BTT was calculated as the percentage of coverage of the target line (i.e., 100 % coverage would imply perfect performance). Specifically, every sampled cursor position was considered to 'cover' the point on the target line with minimal Euclidian distance to the current cursor position. For every trial, the number of unique 'covered' points was divided by the total number of points on the target line and multiplied by 100 [19, 20]. This calculation results in a high accuracy score when the cursor is moved on or parallel to the target line at the same speed as the target dot. In contrast, the score decreases when the cursor is moved too fast or too slowly, when it is moved away from the target line or in the wrong direction, or when cutting corners in the 'zigzag' condition. To derive individual performance indices, accuracy scores were averaged across all 'straight' lines and across all 'zigzag' lines. The mean accuracy of zigzag lines was then used as an indicator of complex motor performance in the analyses.

General procedure

Testing was distributed across two days to prevent fatigue (Supplementary Figure 1). On test session 1, participants received general information before signing the informed consent. Before completing the first three neuropsychological tasks, they underwent MoCA (cognitive functioning) and PPVT (crystallized intelligence [21]) assessments and were administered the BSI-18 (psychological well-being). Participants were also asked to fill in questionnaires regarding lifestyle and medical history, handedness, physical activity, and health-related quality of life. At the end of this session, participants were familiarized with the BTT. Test session 2 comprised the last six neuropsychological tasks and the BTT. The order of the tasks was fixed to minimize between-subject variability in order to avoid such unspecific variance in the latent variable extraction [1]. On the first day, we administered the following neuropsychological tasks: (1) SST, (2) CAST, (3) DST. On the second day, we administered the remaining tasks: (4) COST, (5) KTT, (6) AT, (7) STT, (8) NST, (9) NLT. To ensure that any fatigue or learning effects would affect the three executive domains to a similar extent, the task order was built such that the sums of task-order positions were equal (i.e., 15) for inhibition, shifting, and updating tasks. To illustrate, the summed task-order position for inhibition tasks is 1 (position of SST) + 6 (AT) + 8 (NST) = 15. It was ensured that no two tasks from the same domain were directly following each other, and that tasks from all domains had been completed before another task from any given domain was administered again. In other words, all domains had to be covered n times before a task from any domain could have been administered for the n + 1-th time.

Data analysis

The neuropsychological data were processed in SPSS 26 (IBM, Armonk, NY) to derive the outcome measures (see Neuropsychological Tasks) [1]. BTT data were analyzed in Matlab 2019b (MathWorks, Natick, MA). Data analysis was performed in R 4.0.2 [22] in RStudio 1.3 (RStudio, Boston, MA), relying on the lavaan package version 0.6-7 [23] for structural equation modelling. The dataset and code are available on https://www.osf.io/5v2rz.

Processing of neuropsychological tasks

The RT-based outcome measures (NST, SST, CAST, COST, NLT) were calculated after excluding error trials and trials with premature responses (i.e., RT < 200 ms). For the shifting tasks (CAST, COST, NLT), trials following error trials were discarded, as it cannot be concluded with certainty whether those trials represent switch or repeat trials [1]. For NST, CAST, COST, and NLT, median RTs were extracted for every condition to calculate the desired outcome measure. For SST, the outcome measure was calculated as defined above. For AT, DST, KTT, and STT, outcomes were calculated based on the number (DST, KTT) or proportion (AT, STT) of correct responses.

Next, a pre-defined validity criterion was applied to every task to ensure that only those datasets were entered in the analyses where we had positive evidence that the individual was applying the task instructions. For the antisaccade task (AT), participants were excluded when their performance was indistinguishable from chance level in the prosaccade block. In other words, given that participants were administered 18 prosaccade trials and had a probability of 1/9 for responding correctly by arbitrarily pressing either response key (numbers 1-9), performance was significantly ($p < .05$) distinguishable from chance level when at least five correct responses occurred, as indicated by a binomial test. For the number-Stroop task (NST), participants were excluded when they failed to respond correctly on at least 12 trials in the neutral and the congruent condition, or on at least 21 trials in the incongruent condition (i.e., significantly better than 1/6 correct), as indicated by a binominal test. For the stop-signal task (SST), participants were excluded if they failed to respond correctly to at least 102 go-trials in the mixed

blocks (i.e., significantly better than 50 % correct), as indicated by a binomial test. In addition, participants were excluded if they performed less than 24 and more than 36 stop-trials correctly. In other words, stopping accuracy had to fall between 40 % and 60 % to exclude that participants slowed their responses too much in the mixed blocks, but did not arbitrarily press either response key. For the category-switch task (CAST), participant were excluded when their task performance did not significantly differ from chance level. Binomial tests indicated that at least 22 trials needed to be correctly completed on single-task blocks, and that at least 40 trials needed to be correctly completed in both of the conditions (repeat, switch) in mixed blocks. The same criteria were used for the color-shape task (COST) and the number-letter task (NLT). Note that trial numbers on the COST differed slightly for counterbalancing reasons: participants had to complete at least 17 correct trials on single-task blocks and at least 35 correct trials in both conditions in mixed blocks in the COST. For the digit-span task (DST), participants were excluded when they failed to correctly complete one forward and one backward trial. For the keep track task (KTT), participants were excluded when they failed to recall all words correctly for at least one trial (regardless of the difficulty). For the spatial 2-back task (STT), participants were excluded when they failed to perform significantly above chance level across all blocks. A binomial test indicated that at least 83 trials had to be performed correctly. Supplementary Table 1 gives an overview of the datasets that were excluded as a result of individual-level validity checks. Taken together, only the stop-signal task mandated the exclusion of > 5 % of the sample. This is due to the especially strict criteria with regard to the correct stopping rate between 40 and 60 % that can only be achieved when response slowing in mixed blocks is avoided. After exclusion, 26 complete (i.e., validity criterion for all tasks passed) datasets from young adults and 92 datasets from older adults were retained for further analysis (see Supplementary Figure 2).

To minimize the influence of extreme scores, observations outside 3 *SD* from the respective group mean were replaced by the value at group mean plus (or minus) 3 *SD* [1]. This approach led to the replacement of nine individual values (0.85 % of the data). After this procedure, only the STT outcome in the young adults showed high kurtosis and was therefore arcsine transformed [1]. Measures based on RT were transformed such that higher scores indicated better performance for correlations and structural equation modeling.

REFERENCES

1. Friedman NP, Miyake A, Altamirano LJ, Corley RP, Young SE, Rhea SA, Hewitt JK. Stability and change in executive function abilities from late adolescence to early adulthood: A longitudinal twin study. Dev Psychol. 2016; 52:326–40. https://doi.org/10.1037/dev0000075 PMID:26619323

2. Mathôt S, Schreij D, Theeuwes J. OpenSesame: an open-source, graphical experiment builder for the social sciences. Behav Res Methods. 2012; 44:314–24. https://doi.org/10.3758/s13428-011-0168-7 PMID:22083660

3. Guitton D, Buchtel HA, Douglas RM. Frontal lobe lesions in man cause difficulties in suppressing reflexive glances and in generating goal-directed saccades. Exp Brain Res. 1985; 58:455–72. https://doi.org/10.1007/BF00235863 PMID:4007089

4. Ito TA, Friedman NP, Bartholow BD, Correll J, Loersch C, Altamirano LJ, Miyake A. Toward a comprehensive understanding of executive cognitive function in implicit racial bias. J Pers Soc Psychol. 2015; 108: 187–218. https://doi.org/10.1037/a0038557 PMID:25603372

5. Roberts RJ (Jr), Hager LD, Heron C. Prefrontal cognitive processes: Working memory and inhibition in the antisaccade task. J Exp Psychol Gen. 1994; 123: 374–93. https://doi.org/10.1037/0096-3445.123.4.374

6. MacLeod CM. Half a century of research on the Stroop effect: an integrative review. Psychol Bull. 1991; 109:163–203. https://doi.org/10.1037/0033-2909.109.2.163 PMID:2034749

7. Nweze T, Nwoke MB, Nwufo JI, Aniekwu RI, Lange F. Working for the future: parentally deprived Nigerian Children have enhanced working memory ability. J Child Psychol Psychiatry. 2021; 62:280–88. https://doi.org/10.1111/jcpp.13241 PMID:32302431

8. Stroop JR. Studies of interference in serial verbal reactions. J Exp Psychol. 1935; 18: 643–62. https://doi.org/10.1037/h0054651

9. Logan GD, Cowan WB. On the ability to inhibit thought and action: A theory of an act of control. Psychol Rev. 1984; 91: 295–327. https://doi.org/10.1037/0033-295X.91.3.295

10. van den Wildenberg WP, van Boxtel GJ, van der Molen MW, Bosch DA, Speelman JD, Brunia CH. Stimulation of the subthalamic region facilitates the selection and inhibition of motor responses in Parkinson's disease. J Cogn Neurosci. 2006; 18:626–36. https://doi.org/10.1162/jocn.2006.18.4.626 PMID:16768365

11. Lange F, Dewitte S. Cognitive flexibility and pro-environmental behaviour: A multimethod approach. Eur J Personal. 2019; 33: 488–505. https://doi.org/10.1002/per.2204

12. Mayr U, Kliegl R. Task-set switching and long-term memory retrieval. J Exp Psychol Learn Mem Cogn. 2000; 26:1124–40. https://doi.org/10.1037//0278-7393.26.5.1124 PMID:11009248

13. Miyake A, Emerson MJ, Padilla F, Ahn JC. Inner speech as a retrieval aid for task goals: the effects of cue type and articulatory suppression in the random task cuing paradigm. Acta Psychol (Amst). 2004; 115:123–42. https://doi.org/10.1016/j.actpsy.2003.12.004 PMID:14962397

14. Rogers RD, Monsell S. Costs of a predictible switch between simple cognitive tasks. J Exp Psychol Gen. 1995; 124: 207–31. https://doi.org/10.1037/0096-3445.124.2.207

15. Gustavson DE, Panizzon MS, Franz CE, Friedman NP, Reynolds CA, Jacobson KC, Xian H, Lyons MJ, Kremen WS. Genetic and environmental architecture of executive functions in midlife. Neuropsychology. 2018; 32:18–30. https://doi.org/10.1037/neu0000389 PMID:28857599

16. Yntema DB. Keeping track of several things at once. Hum Factors. 1963; 5:7–17. https://doi.org/10.1177/001872086300500102 PMID:14002586

17. Friedman NP, Miyake A, Young SE, DeFries JC, Corley RP, Hewitt JK. Individual differences in executive functions are almost entirely genetic in origin. J Exp Psychol Gen. 2008; 137:201–25. https://doi.org/10.1037/0096-3445.137.2.201 PMID:18473654

18. Sisti HM, Geurts M, Clerckx R, Gooijers J, Coxon JP, Heitger MH, Caeyenberghs K, Beets IA, Serbruyns L, Swinnen SP. Testing multiple coordination constraints with a novel bimanual visuomotor task. PLoS One. 2011; 6:e23619. https://doi.org/10.1371/journal.pone.0023619 PMID:21858185

19. King BR, van Ruitenbeek P, Leunissen I, Cuypers K, Heise KF, Santos Monteiro T, Hermans L, Levin O, Albouy G, Mantini D, Swinnen SP. Age-Related Declines in Motor Performance are Associated With Decreased Segregation of Large-Scale Resting State Brain Networks. Cereb Cortex. 2018; 28:4390–402. https://doi.org/10.1093/cercor/bhx297 PMID:29136114

20. Zivari Adab H, Chalavi S, Monteiro TS, Gooijers J, Dhollander T, Mantini D, Swinnen SP. Fiber-specific variations in anterior transcallosal white matter structure contribute to age-related differences in motor performance. Neuroimage. 2020; 209:116530. https://doi.org/10.1016/j.neuroimage.2020.116530 PMID:31931154

21. Horn JL, Cattell RB. Age differences in fluid and crystallized intelligence. Acta Psychol (Amst). 1967; 26:107–29. https://doi.org/10.1016/0001-6918(67)90011-x PMID:6037305

22. R Core Team. R: A language and environment for statistical computing. Vienna, Austria: R Foundation for Statistical Computing. 2020. Available from: https://www.R-project.org/

23. Rosseel Y. lavaan: An R package for structural equation modeling. J Stat Softw. 2012 ; 48. Available from: http://www.jstatsoft.org/v48/i02/ https://doi.org/10.18637/jss.v048.i02
