# Supplementary material for: Cognition and action: a latent variable approach to study contributions of executive functions to motor control in older adults
Source: Aging (Albany NY). 2021 Jun 24;13(12):15942–63. doi: 10.18632/aging.203239 (PMC8266336; doi:10.18632/aging.203239)
Supplement: Supplementary Figures [file aging-13-203239-s002.pdf]

SUPPLEMENTARY FIGURES

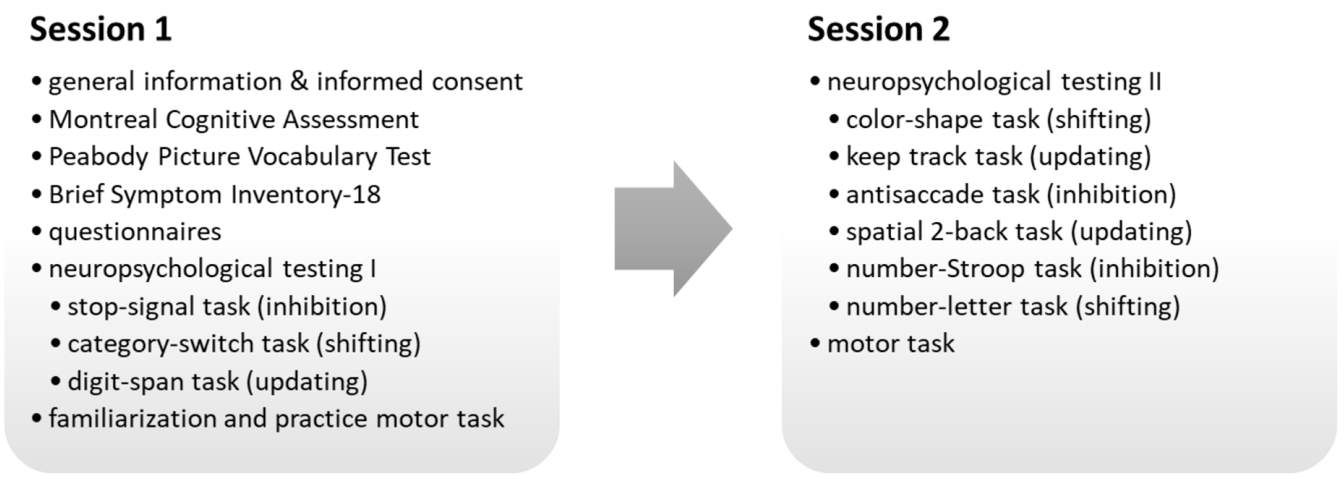

Supplementary Figure 1. Description of the study protocol.

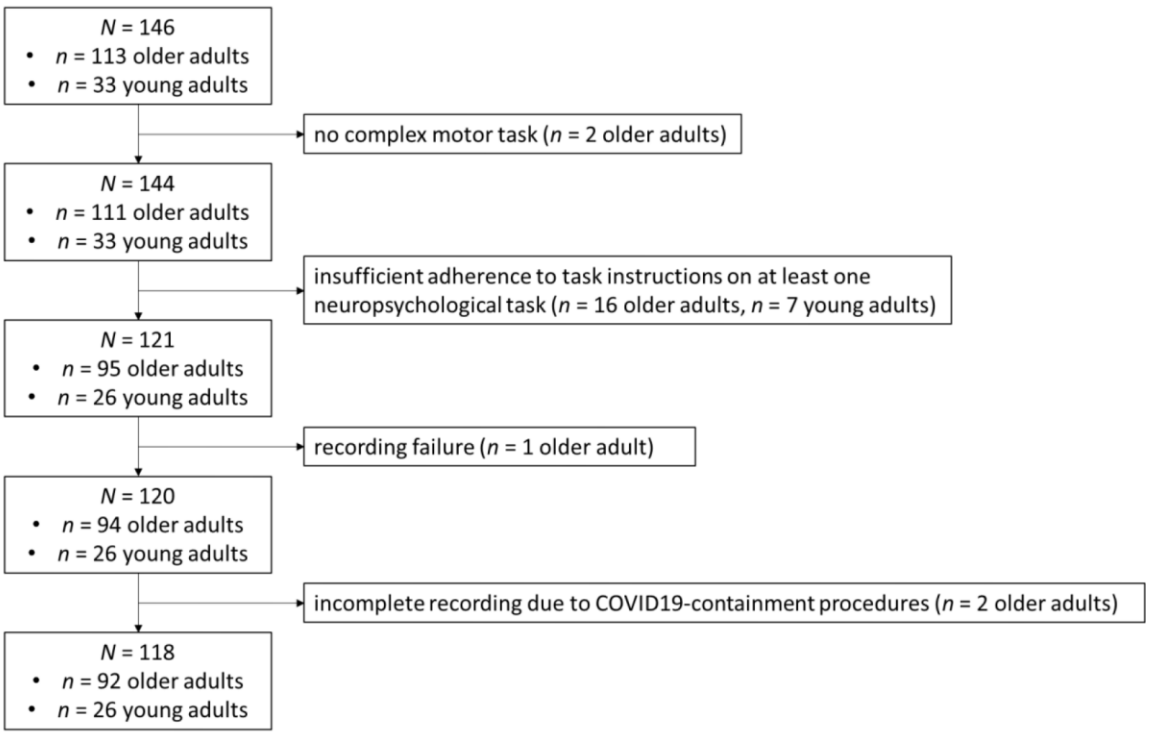

Supplementary Figure 2. Flow-chart describing the selection of the sample for the current analyses.
