# Supplementary material for: Cognition and action: a latent variable approach to study contributions of executive functions to motor control in older adults
Source: Aging (Albany NY). 2021 Jun 24;13(12):15942–63. doi: 10.18632/aging.203239 (PMC8266336; doi:10.18632/aging.203239)
Supplement: Supplementary Table 1 [file aging-13-203239-s003.pdf]

SUPPLEMENTARY TABLE

Supplementary Table 1. Datasets available for analysis after application of validity criteria.

Task	Group	*n* recorded	*n* (%) excluded	*n* available for analysis
Inhibition				
Antisaccade task	older adults	109	0 (0.00 %)	109
	young adults	33	0 (0.00 %)	33
Number-Stroop task	older adults	108	0 (0.00 %)	108
	young adults	33	0 (0.00 %)	33
Stop-signal task	older adults	110	7 (6.36 %)	103
	young adults	33	7 (21.21 %)	26
Shifting				
Category-switch task	older adults	111	1 (0.09 %)	110
	young adults	33	0 (0.00 %)	33
Color-shape task	older adults	109	4 (3.67 %)	105
	young adults	33	1 (3.03 %)	32
Number-letter task	older adults	108	2 (1.85 %)	106
	young adults	33	0 (0.00 %)	33
Updating				
Digit-span task	older adults	111	2 (1.80 %)	109
	young adults	33	0 (0.00 %)	33
Keep track task	older adults	109	0 (0.00 %)	109
	young adults	33	0 (0.00 %)	33
Spatial 2-back task	older adults	107	2 (1.87 %)	105
	young adults	33	0 (0.00 %)	33
